# Canonical Wnt signalling regulates epithelial patterning by modulating levels of laminins in zebrafish appendages

**DOI:** 10.1242/dev.118703

**Published:** 2015-01-15

**Authors:** Monica Nagendran, Prateek Arora, Payal Gori, Aditya Mulay, Shinjini Ray, Tressa Jacob, Mahendra Sonawane

**Affiliations:** 1 Department of Biological Sciences, Tata Institute of Fundamental Research, Colaba, Mumbai 400005, India; 2 Indian Institute of Science Education and Research, Pune 411008, India

**Keywords:** Appendages, Epithelial cell shapes, Laminin, Wnt signalling, Zebrafish, Median fin fold

## Abstract

The patterning and morphogenesis of body appendages – such as limbs and fins – is orchestrated by the activities of several developmental pathways. Wnt signalling is essential for the induction of limbs. However, it is unclear whether a canonical Wnt signalling gradient exists and regulates the patterning of epithelium in vertebrate appendages. Using an evolutionarily old appendage – the median fin in zebrafish – as a model, we show that the fin epithelium exhibits graded changes in cellular morphology along the proximo-distal axis. This epithelial pattern is strictly correlated with the gradient of canonical Wnt signalling activity. By combining genetic analyses with cellular imaging, we show that canonical Wnt signalling regulates epithelial cell morphology by modulating the levels of laminins, which are extracellular matrix components. We have unravelled a hitherto unknown mechanism involved in epithelial patterning, which is also conserved in the pectoral fins – evolutionarily recent appendages that are homologous to tetrapod limbs.

## Abstract (precis)

**Highlighted article**: In the zebrafish fin, a Wnt gradient dictates the expression of laminin α5, which signals via integrin α3 to control epithelial cell morphology.

## INTRODUCTION

Vertebrate appendages display a remarkable diversity in structure and function. The evolution of appendages such as fins and subsequently limbs influenced the modes of locomotion and animal behaviours. Evolutionarily, the occurrence of median fins precedes that of paired fins (Freitas et al., 2006). Experimental and fossil evidences support the hypothesis that mechanisms governing the development of paired appendages (paired fins and limbs) originated in the more ancestral median fin (Coates, 1994; Freitas et al., 2006; Shubin et al., 1997).

Zebrafish possesses a continuous median fin and paired pectoral fins during its late embryonic and larval stages. Similar to vertebrate limb development, the outgrowth of both the pectoral and median fins is maintained by an epithelial structure – the apical ectodermal ridge (AER). The morphogenesis of the median fin is initiated at 16 hpf with the formation of the apical ectodermal ridge (AER) along the dorsal and ventral midline of the zebrafish embryo (Dane and Tucker, 1985). The fin AER is formed by the folding of a bi-layered epidermis consisting of basal epithelial cells and an outer periderm (Dane and Tucker, 1985). Unlike limb development, where the AER eventually regresses, during fin development – whether median or pectoral – the AER grows to become an apical ectodermal fold or AEF (Webb et al., 2007; Yano et al., 2012). Despite some differences, both AER and AEF act as key signalling centres that coordinate appendage morphogenesis (Fernandez-Teran and Ros, 2008; Mercader, 2007). The proper formation and maintenance of the AER and AEF is crucial for subsequent events of appendage development (Webb et al., 2007; Yano et al., 2012).

Canonical Wnt ligands play key roles during limb development. Studies in chick have shown that Wnt2b and Wnt8c regulate the expression of Fgf10 in the lateral plate mesoderm, which is essential for the AER induction (Kawakami et al., 2001). Furthermore, Wnt3/3a-mediated canonical Wnt signalling in the limb epithelium is essential for the induction of Fgf8 expression and AER formation in mouse and chick (Barrow et al., 2003; Fernandez-Teran and Ros, 2008; Galceran et al., 1999; Kawakami et al., 2001; Kengaku et al., 1998; Soshnikova et al., 2003). Consistently, mutations in the *WNT3* gene in humans affect limb development, resulting in tetra-amelia (Niemann et al., 2004). In *Drosophila*, the role of canonical Wingless (Wg) signalling during appendage development has been well studied (Baker, 1988a,b; Couso et al., 1993; Simox et al., 1989). It regulates the size of apical cell circumference and changes in cellular morphology in *Drosophila* wing imaginal disc epithelium (Jaiswal et al., 2006; Widmann and Dahmann, 2009).

The importance of epithelial integrity in appendage development is underscored by the fact that loss of AER integrity leads to limb defects (Miner et al., 1998). Extracellular matrix (ECM) components are crucial for maintenance of epithelial integrity. Laminins are key ECM molecules that form a heterotrimeric complex of α, β and γ chains, and are crucial for cell matrix adhesion and signalling. Studies in *Drosophila* have shown that mutations in laminin chains results in blister formation in the wing epithelium (Henchcliffe et al., 1993; Martin et al., 1999; Urbano et al., 2009). In the case of vertebrates, loss of laminin α5 causes limb abnormalities in mice and fin deformities in zebrafish larvae (Miner et al., 1998; Webb et al., 2007). Besides laminin α5, large-scale mutagenesis screens in zebrafish have identified the ECM components Fras1, Frem1, Frem2 and hemicentin 1 (Hmcn1) to be essential for the proper development of larval fin appendages (Carney et al., 2010; van Eeden et al., 1996). Despite having a fair understanding of the involvement of ECM molecules in appendage development, the regulation of their synthesis is largely unexplored.

We set out to explore whether epithelial patterning exists in vertebrate appendages and how this pattern is established during embryonic development. We used a simple appendage – the median fin in the zebrafish embryo to address this issue. Being an evolutionarily old unpaired appendage, it offers a unique opportunity to investigate ancient mechanisms involved in epithelial patterning. We show that a gradient of canonical Wnt signalling activity controls epithelial cell morphologies across the PD axis by regulating expression of laminins. This mechanism involved in patterning the median fin fold epithelium is conserved in the course of evolution to pattern pectoral fins.

## RESULTS

### Epithelial cell shape pattern correlates with the canonical Wnt signalling gradient across the proximo-distal axis in developing median fin

**Fig. 1.**
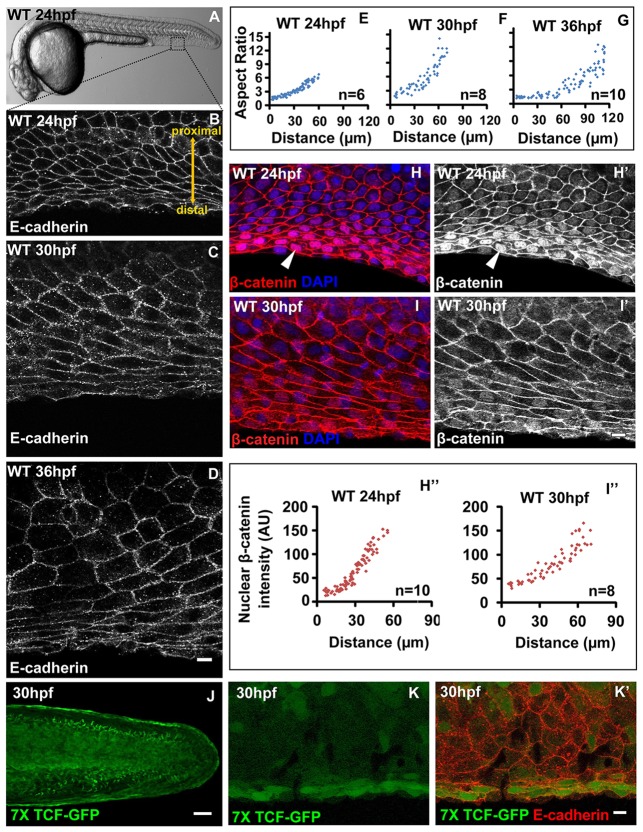
**Correlation between cellular pattern and Wnt signalling gradient in the median fin fold epithelium.** (A) Bright-field image of 24 hpf wild-type zebrafish embryo. The dotted box represents the region of the fin epithelium imaged by confocal microscopy. (B-G) E-cadherin staining and aspect ratio plots of median fin epithelial cells at 24 (B,E), 30 (C,F) and 36 hpf (D,G) in wild-type embryos. Aspect ratios are plotted against the distance of the cells from the base of the fin fold along PD axis. (H-I′) β-Catenin-DAPI overlays (H,I) and β-catenin staining (H′,I′) and its quantification (H″,I″) in median fin epithelium at 24 hpf (H-H′) and 30 hpf (I-I′). (J-K′) *Tg(7XTCF-Xia.Siam:GFP)ia^4^* line showing GFP expression in the distal cells of the median fin at lower magnification (J) and at higher magnification (K,K′) along with E-cadherin staining. Arrowheads in H,H′ indicate nuclear β-catenin. Scale bars: 10 µm B-D,H-I′,K,K′; 50 µm in J. AU, arbitrary units.

In mouse and chick, the epithelial cells in the apical ectodermal ridge (AER) exhibit cellular morphologies distinct from the rest of the appendage epithelium (Fernandez-Teran and Ros, 2008). We asked whether the median fin epithelium of zebrafish embryos shows any patterning at the cellular level. We analysed the shapes of peridermal cells as well as basal epithelial cells (supplementary material Fig. S1A) in the median fin epithelium of embryos 24-36 hours post fertilization (hpf) by staining for E-cadherin, followed by estimation of aspect ratios. The aspect ratio is indicative of the extent of elongation: the higher the aspect ratio, the higher is the cell elongation along one axis. Our analysis revealed that the basal cells towards the distal side of the fin epithelium (but not the peridermal cells) are more elongated along the antero-posterior axis when compared with the proximal cells (Fig. 1A-D; supplementary material Fig. S1C-D′). The distal cells gradually acquire stretched morphology, resulting in an increase in the aspect ratio from 6-7 at 24 hpf to 10-12 at 36 hpf. By contrast, cells towards the proximal side retain a polygonal morphology during this time window (Fig. 1E-G). In addition, orthogonal sections of confocal images revealed that the apical-basal height of the basal cells decreases and the sub-epidermal space is formed from 20 hpf to 30 hpf. Although there is no obvious pattern in cell height along the PD axis at 30 hpf, distal cells are taller when compared with the proximal cells at 20 hpf (supplementary material Fig. S1B). Thus, initially taller distal cells at 20 hpf become flat and acquire stretched morphology along the antero-posterior axis during subsequent median fin fold morphogenesis. As peridermal cells do not show changes in morphology along the PD axis, henceforth the term ‘epithelial patterning’ is used in the context of basal epithelial cells.

Since canonical Wnt signalling plays an important role in limb morphogenesis, we asked whether it would pattern the median fin epithelium. Immunolocalisation revealed the beginning of nuclear accumulation of β-catenin in the basal cells of median fin epithelium at 20 hpf, indicating activation of Wnt signalling (supplementary material Fig. S1E,E′). Its accumulation peaks at 24 hpf and gradually decreases by 36 hpf (Fig. 1H-I′; data not shown). Our quantification revealed that between 24 and 30 hpf the levels of nuclear β-catenin increase exponentially towards the distal end as a function of distance along the PD axis (Fig. 1H″,I″).

To further confirm Wnt signalling activity in the fin, we examined embryos from a transgenic Wnt reporter line *Tg(7*×*TCF-Xla.Siam:GFP)^ia^^4^* (Moro et al., 2012). We observed higher expression of GFP in the distal stretched cells of the fin epithelium correlating with the nuclear β-catenin levels (Fig. 1J-K′). From the previous analysis, there are indications that *frizzled 2* (*fzd2*) is expressed in the growing median fin fold (Nikaido et al., 2013). In addition, Wnt3 and Wnt3a are known to be expressed in the AER of mouse and chick, respectively (Galceran et al., 1999; Kawakami et al., 2001). Our *in situ* hybridisation analysis revealed that *wnt3a* is expressed as early as 16 hpf in the developing median fin fold, whereas the expression of both *wnt3a* and *fzd2* appeared more restricted to the distal domain of the fin fold at 24 and 30 hpf (supplementary material Fig. S1F-J′). To conclude, there is a correlation between the cell shape changes and the extent of canonical Wnt signalling activity across the PD axis of the median fin epithelium.

### Modulation of canonical Wnt signalling gradient dictates changes in epithelial cell shape pattern in the median fin epithelium

**Fig. 2.**
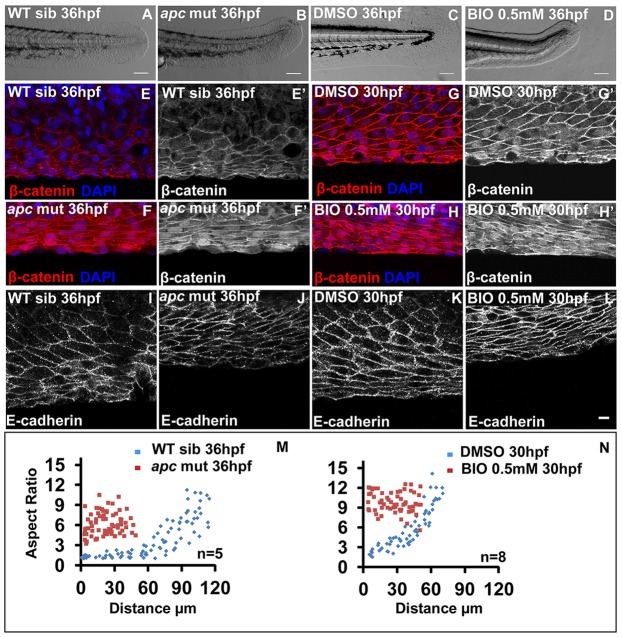
**Gain of Wnt signalling results in acquisition of stretched cell morphology in the median fin fold.** Bright-field images of wild-type sibling (A), *apc* mutant (B), DMSO- (C) and 0.5 mM BIO-treated (D) embryos. (E-H′) β-Catenin-DAPI overlays (E-H) and β-catenin staining (E′-H′) in given genetic backgrounds and treatments. (I-L) E-cadherin staining in wild-type siblings (I), *apc* mutant (J), DMSO control (K) and BIO-treated (L) embryos. (M,N) Comparison of epithelial cell aspect ratio plots between sibling and *apc* mutant (M) as well as between DMSO and BIO-treated embryos (N). Scale bars: 0.1 mm in A-D; 10 µm in E-L.

We observed a correlation between the extent of canonical Wnt signalling activity and cell elongation in the fin epithelium. We manipulated the levels of Wnt signalling to test whether it plays a role in patterning the median fin epithelium. For gain-of-function studies, we used *apc* mutant (Paridaen et al., 2009) and a chemical inhibitor of GSK-3β called BIO (Gore et al., 2011); both APC and GSK3β are components of the β-catenin destruction complex (Dominguez et al., 1995; He et al., 1995; Polakis, 2000; Rubinfeld et al., 1996). In both *apc* mutant and BIO-treated embryos, the median fin fold shows an overall reduction at 36 hpf (Fig. 2A-D). We observed an appreciable increase in nuclear β-catenin in the fin epithelium of *apc* mutants, as well as in BIO-treated embryos at 36 hpf and 30 hpf, respectively (Fig. 2E-H′). Consistently, genotyped *apc* mutant embryos at early stages showed an increase in nuclear β-catenin levels in the fin epithelium (supplementary material Fig. S2A-F′). Further cell shape analysis revealed that the epithelial cells throughout the PD axis of the fin fold exhibit stretched morphology by 36 hpf in *apc* mutants and by 30 hpf in BIO-treated embryos (Fig. 2I-N).

To test whether the effect on cell morphology is tissue autonomous, we performed a localised treatment of the BIO drug. We soaked sephacryl beads in BIO and placed them close to the ventral fin of zebrafish larvae immobilised in agarose. In comparison with control, the BIO-soaked beads produced a local decrease in expansion of the fin region (supplementary material Fig. S3A,B). Further analysis revealed that cells in this region of the fin fold exhibit high levels of nuclear β-catenin and stretched morphology (supplementary material Fig. S3C-D′).

**Fig. 3.**
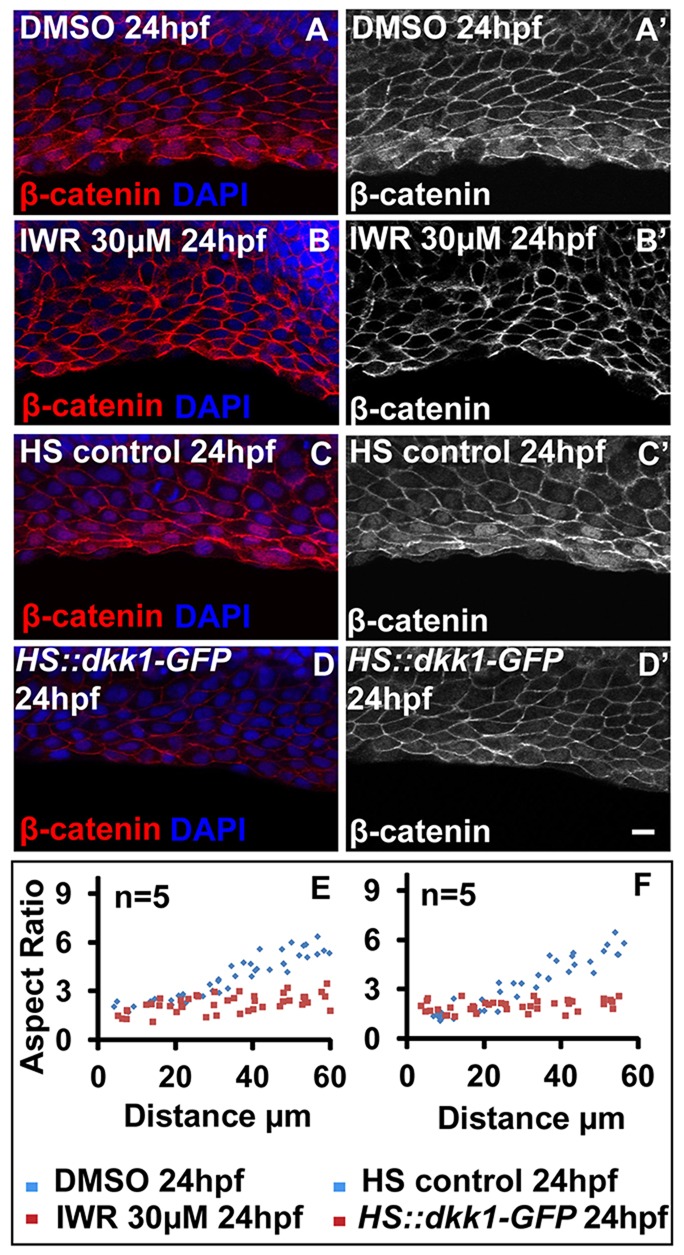
**Loss of Wnt signalling results in polygonal morphology throughout the median fin epithelium.** (A-D′) β-Catenin-DAPI overlays (A-D) and β-catenin staining (A′-D′) in DMSO- (A,A′), IWR-treated (B,B′), heat-shock control (C,C′) and *HS:dkk1-GFP* embryos (D,D′) at 24 hpf. (E,F) Comparison of aspect ratios between DMSO and IWR-treated (E), and heat-shock control and *HS:dkk1-GFP* embryos (F). Scale bar: 10 µm.

To test the effect of decreased Wnt signalling on fin epithelium, we used a heat shock-inducible Wnt inhibitor line *HS:dkk1-GFP* (Stoick-Cooper et al., 2007) and IWR (inhibitor of Wnt response) (Chen et al., 2009). Dickkopf is a secreted Wnt inhibitor that antagonises Wnt signalling by interacting with the co-receptor Lrp6 (Mao et al., 2001) whereas IWR stabilises the destruction complex, promoting degradation of β-catenin (Chen et al., 2009). Pulsed heat shock of *HS:dkk1-GFP* embryos between 20 hpf and 24 hpf or treatment of wild-type embryos with 30 µM IWR at 16 hpf followed by analysis at 50 hpf did not reveal any significant change in fin morphology (supplementary material Fig. S3E-H,K,L). However, both the treatments resulted in a significant decrease in nuclear β-catenin levels at 24 hpf when compared with their respective controls (Fig. 3A-D′). The aspect ratio analysis revealed that, unlike the control embryos, in heat-shocked and IWR-treated embryos the distal cells display low aspect ratio or polygonal morphology (Fig. 3E,F). The longer treatments with IWR and heat-shock treatments beyond 24 hpf yielded inconclusive results. This is presumably due to high amplitude of Wnt signalling at and around 24 hpf, which is not dampened effectively by IWR treatment and by heat shock. In addition, either continuous heat shock from 20 hpf to 24 hpf or longer heat shock treatments resulted in deleterious effects on the embryos, including retarded growth and embryonic death (supplementary material Fig. S3I,J; data not shown).

We further analysed the effect of gain and loss of Wnt signalling on the heights of basal epithelial cells in the median fin fold. In BIO-treated embryos, the distal cells continued to remain columnar at 30 hpf and the overall apical-basal height of epithelial cells throughout the PD axis is higher compared with the control embryos (supplementary material Fig. S4E,F). Similarly, the *apc* mutant embryos displayed marginally taller proximal cells when compared with siblings (supplementary material Fig. S4A,B). However, there is no significant alteration in cell heights upon loss of Wnt signalling (supplementary material Fig. S4G,H). Interestingly, in both *apc* mutants and in BIO-treated embryos there is an obvious effect on the formation of sub-epidermal space (supplementary material Fig. S4A-F), which is required for organisation of ECM in the median fin (Feitosa et al., 2012).

We conclude that differential canonical Wnt signalling along the PD axis is essential for acquisition of the epithelial cell shape pattern in the median fin fold. Furthermore, higher Wnt signalling results in delay in apical-basal flattening of the fin epithelial cells and interferes in formation of sub-epidermal space, suggesting major effect on fin morphogenesis. For gaining mechanistic insights, we focussed on analysing polygonal and stretched morphologies of the basal cells as they are robustly modulated by both gain and loss of Wnt signalling along the PD axis.

### Extent of canonical Wnt signalling and its persistence within a specific time window regulates patterning of the median fin epithelium

**Fig. 4.**
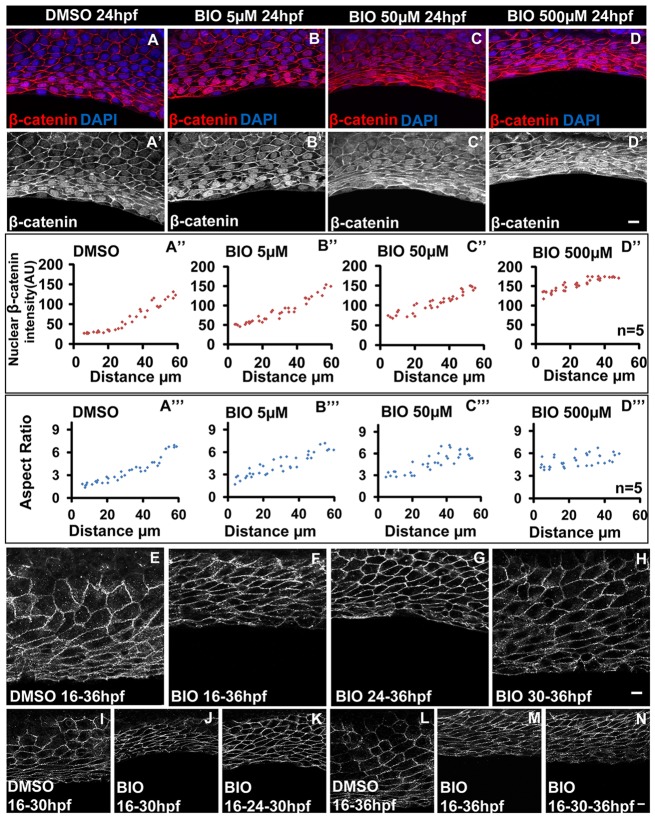
**Canonical Wnt signalling is required persistently within a specific time window to alter cell shapes.** (A-D′) β-Catenin-DAPI overlays (A-D) and β-catenin staining (A′-D′) in DMSO control (A,A′) and embryos treated with the mentioned doses of BIO drug (B-D,B′-D′). (A″-D‴) Comparison of nuclear β-catenin levels (A″-D″) and aspect ratio plots (A‴-D‴) for DMSO control and embryos treated with the mentioned doses of BIO. (E-H) E-cadherin staining in embryos treated with DMSO (E) and with 0.5 mM BIO at 16 (F), 24 (G) and 30 (H) hpf. (I,J,L,M) Cell-shape analysis in DMSO control (I,L) and embryos treated with BIO from 16 hpf continuously and analysed at 30 (J) and at 36 (M) hpf. (K,N) Embryos in which the drug was (K) washed off at 24 hpf and cell shape analysis carried out at 30 hpf or (N) washed off at 30 hpf and analysed at 36 hpf. Scale bars: 10 µm. AU, arbitrary units.

We showed that Wnt signalling is essential for epithelial patterning in the median fin fold. However, it was not clear whether the extent of Wnt signalling controls graded changes in cellular morphology along the PD axis. To address this, wild-type embryos were treated with different concentrations of BIO at 16 hpf and cell shapes were analysed at 24 hpf. We reasoned that the effect of BIO will be more pronounced in the proximal cells that otherwise normally have low nuclear β-catenin levels and polygonal morphology. Indeed, the quantification revealed that as the concentration of BIO increases, the levels of nuclear β-catenin increases in the proximal cells (Fig. 4A-D′), flattening out the intensity curve, which shows an exponential trend in the control embryos (Fig. 4A″-D″). Further analysis of aspect ratios showed that there is a strong correlation between increased nuclear β-catenin levels in the proximal cells and the extent of cell elongation (Fig. 4A‴-D‴). These data indicate that proximal cells, which are fated to become polygonal, respond to the increased canonical Wnt signalling activity and acquire a stretched morphology.

To check whether Wnt signalling influences epithelial cell shape changes in a defined time window, BIO was added at different stages of development and cell shapes were analysed at 36 hpf. Zebrafish embryos treated from 16 hpf to 36 hpf showed stretched cell morphology across the entire PD axis of the median fin epithelium (Fig. 4E,F). BIO treatment starting from 24 hpf resulted in milder stretching of the cells on the proximal side (Fig. 4G), whereas treatments starting at 30 and 36 hpf and analysed at 36, 48 and 60 hpf did not influence the proximal cells to acquire a stretched morphology (Fig. 4H, data not shown). Furthermore, BIO treatment did not lead to accumulation of nuclear β-catenin in the basal epithelial cells after 30 hpf (supplementary material Fig. S4I-J′). These data suggest that beyond 30 hpf Wnt signalling cannot be modulated and hence the cell shape pattern in the median fin fold epithelium does not change.

To test whether persistent Wnt signalling is required between 16 and 30 hpf or whether a short pulse is sufficient to influence cellular morphology, we treated the zebrafish embryos with BIO at 16 hpf. Some embryos were retained in the drug and the drug was washed off at 24 or 30 hpf in the rest. Cell shapes were then analysed at 30 and/or 36 hpf. The embryos in which the drug was washed off at 24 hpf showed an intermediate morphology of the fin epithelial cells. By contrast, those washed off at 30 hpf or treated continuously up to 36 hpf showed stretched cell morphology throughout the fin epithelium (Fig. 4I-N). Our results demonstrate that the persistence of Wnt signalling from 16 to 30 hpf and its extent regulate epithelial cell morphology along the PD axis of the median fin epithelium.

### Cell proliferation and changes in cell numbers do not influence epithelial pattern in the median fin epithelium

**Fig. 5.**
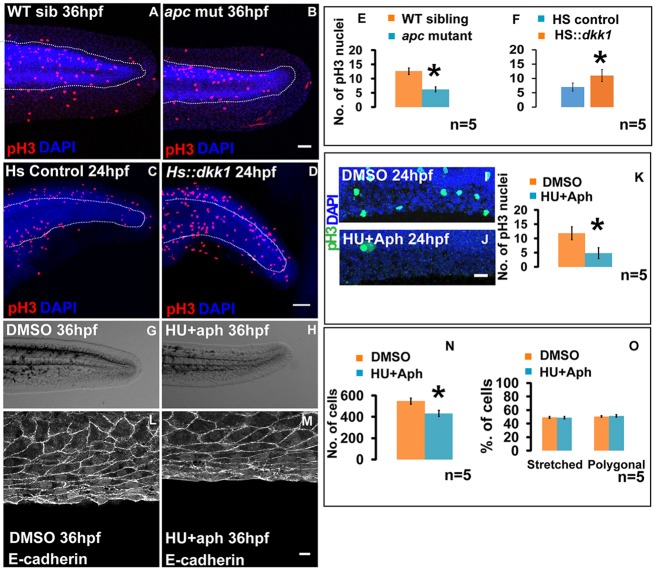
**Patterning in the median fin epithelium is not influenced by proliferation rates and total cell numbers.** Phospho-histone 3 and DAPI staining under given genetic conditions or treatments at 24 hpf (A-D,I,J). (E,F,K) Quantification reveals decrease in proliferation in *apc* mutant (E) and drug (HU+aph) treated (K) embryos, and an increase in *HS:dkk1-GFP* embryos (F) when compared with their respective controls. (G,H) Median fin morphology at 36 hpf in DMSO- (G) and HU+aph-treated embryos (H). (L,M) Cellular morphology in 36 hpf control (L) and HU+aph-treated (M) embryos. (N) Comparison of total number of cells in the fin fold of DMSO control and HU+aph-treated embryos. Proportions of stretched and polygonal cells in control and HU+aph-treated embryos at 36 hpf (O). Scale bars: 50 µm in A-D,I,J; 10 µm in L,M. *P<0.01 (Student's *t*-test).

The median fin fold is a growing epithelial structure. In such a growing epithelium, differential cell proliferation may influence the morphology of cells (Mao et al., 2013). We asked whether the Wnt signalling affects cell morphology through its effect on cell proliferation. Phospho-histone3 staining, which labels mitotic cells, revealed that in the median fin of *apc* mutant, proliferation is decreased by 51% (*P*<0.001; Fig. 5A,B,E). By contrast, *HS:dkk1-GFP* heat-shocked embryos showed a 36% increase in proliferation (*P*<0.05; Fig. 5C,D,F). This raised a possibility that the altered cellular morphology seen in the fin epithelium of *apc* mutant could be a consequence of reduced proliferation. To test this, we treated 18 hpf wild-type zebrafish embryos with 20 mM hydroxyurea (HU) and 150 µM aphidicolin, which resulted in a significant decrease in the overall size of the fin fold at 36 hpf (Fig. 5G,H). These concentrations effectively decrease cell proliferation by 60% in the fin folds as early as 24 hpf (*P*<0.01; Fig. 5I-K). Despite a significant decrease in cell number (*P*<0.001; Fig. 5N), HU and aphidicolin-treated embryos at 36 hpf exhibited approximately similar proportions of stretched and polygonal cells as seen in the fin epithelium of control embryos (Fig. 5L,M,O). These results strongly argue against the possibility that the regulation of cellular morphology in the median fin epithelium by Wnt signalling is due to reduced proliferation and cell numbers.

### Laminin α5: a downstream target of canonical Wnt signalling regulates cellular morphology across the PD axis of median fin epithelium

**Fig. 6.**
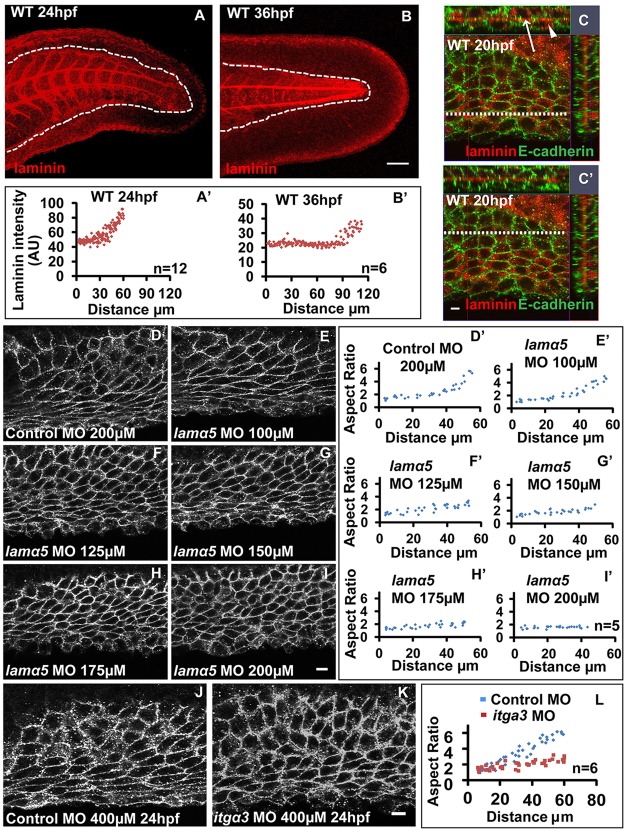
**Laminin α5 regulates cellular patterning in the median fin fold.** Maximum intensity projections of laminin staining and plot for laminin intensities across the PD axis at 24 (A,A′) and 36 (B,B′) hpf. (C,C′) Laminin and E-cadherin staining in the fin-fold of 20 hpf embryo. Orthogonal sections (along the *y*-axis and *x*-axis; white dotted lines in C and C′ represent where the section along the *x*-axis is taken) are for the distal (C) and proximal (C′) parts of the median fin fold epithelium. (D-I′) Analysis of cell shape in the median fin fold of embryos at 30 hpf injected with control (D) and indicated doses of *lama5* morpholino (E-I) and the corresponding aspect ratios along the PD axis (D′-I′). (J-L) In comparison with control (J), distal cells in the fin fold of *itga3* morphants (K) show polygonal morphology, as revealed by the aspect ratio plot (L). Scale bars: 50 µm in A,B; 10 µm in C-K). Arrow and arrowhead in C indicate extracellular and intracellular laminin, respectively. AU, arbitrary units.

Extracellular matrix (ECM) plays an important role in several morphogenetic events, including appendage development in both invertebrates and vertebrates (Miner and Yurchenco, 2004; Rozario and DeSimone, 2010). As cell-matrix adhesion is known to regulate cell shapes (Berrier and Yamada, 2007; Boudreau and Jones, 1999; Rozario and DeSimone, 2010), we analysed the distribution of laminins – key ECM molecules – in the median fin fold. Immunostaining using a pan-laminin antibody at 24 and 36 hpf (Fig. 6A,B) revealed an increase in levels of laminins from proximal to distal regions (Fig. 6A′,B′). Laminin immunostaining at 20 hpf revealed that there are higher levels of intracellular laminins on the distal side compared with the proximal side (Fig. 6C,C′). This observation further suggests that fin fold epithelial cells synthesise and secrete laminins to form the basement membrane.

Laminins are hetero-trimeric molecules assembled from α, β and γ chains. Laminin α5, a component of the laminin 511 complex, plays an essential function during vertebrate appendage development (Miner et al., 1998; Webb et al., 2007). We asked whether laminin α5 is involved in regulation of cell shapes. We reasoned that if the levels of laminin influence cell shapes, then gradual knockdown of laminin α5 (*lama5*) should gradually alter cell morphologies. We injected increasing concentrations of *lama5* splice-site morpholino (Webb et al., 2007) and analysed cellular morphology and aspect ratios at 30 hpf. An increase in morpholino concentrations resulted in increased proportions of unspliced transcripts and overall reduction in *lama5* transcripts levels (supplementary material Fig. S5A-C). As the dose of the morpholino increased, the graded changes in the cellular morphology across the PD axis are gradually lost (Fig. 6D-I). At the highest morpholino concentration, the overall size of the fin was reduced (supplementary material Fig. S6F,G), as reported earlier (Webb et al., 2007), and the cells on the distal side of the median fin epithelium showed low aspect ratios and polygonal morphology (Fig. 6D′-I′). In addition, upon knockdown of *lama5*, the proximal cells in the fin appear polygonal but smaller when compared with the proximal cells in control embryos. However, there was no effect on the levels of nuclear β-catenin in the *lama5* morphants (supplementary material Fig. S6A-E). These data suggest that having high levels of laminin α5 on the distal side of the median fin epithelium would be crucial to acquire a stretched morphology.

Laminins act as ligands for integrins. Besides cell-matrix adhesion, this receptor-ligand interaction is also essential for signalling (Boudreau and Jones, 1999). Laminin α5 is known to interact with either integrin α3β1 or α6β1 (Colognato and Yurchenco, 2000). In zebrafish, integrin α3 (*itga3*) is expressed in the median fin and is required for fin morphogenesis (Carney et al., 2010). Hence, we asked whether laminin α5 regulates cellular morphology through its interaction with integrin α3β1. Our results show that at 24 hpf, the distal cells begin to acquire a stretched morphology in the control morphants. However, in *itga3* morphants they continue to exhibit polygonal morphology (Fig. 6J-L). This cellular phenotype is transient, possibly owing to functional redundancy between the integrins. The similarity between *itga3* and *lamα5* knockdown phenotypes suggest that they act in the same pathway and that their interaction is crucial for regulating cellular morphology in the median fin epithelium.

**Fig. 7.**
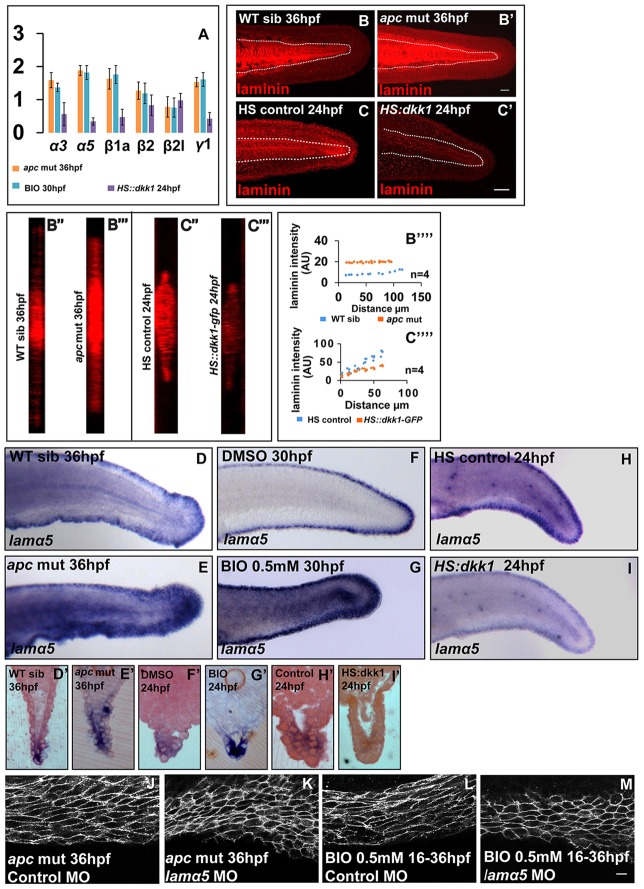
**Canonical Wnt signalling regulates the levels of laminins in the median fin fold.** (A) Graph showing transcript level changes of laminins under gain and loss of canonical Wnt signalling by qRT-PCR. (B-C⁗) Maximum intensity projections of laminin staining in the fin fold of sibling (B), *apc* mutant (B′), HS control (C) and HS::*dkk-GFP* (C′) embryos, their respective orthogonal sections (B″,B‴,C″,C‴) and plots comparing laminin intensities across PD axis in given genetic conditions (B⁗,C⁗). (D-I′) *In situ* hybridisation using *lama5* probes in given genetic backgrounds or treatments (D-I) and sections of stained embryos (D′-I′). (J-M) Cell shape analysis in *apc* mutant (J,K) and 0.5 mM BIO-treated embryos (L,M) injected with control (J,L) or *lama5* morpholino (K,M). The extent of cell stretching is reduced in the absence of *lama5* function (K,M). Scale bars: 50 µm in B-C′; 10 µm in J-M.

A correlation between the Wnt signalling activity, epithelial cell shape and graded laminin deposition in the median fin fold prompted us to hypothesise that Wnt signalling regulates epithelial cell morphology by modulating laminin synthesis. To test the hypothesis, we compared transcript levels of fin fold-specific laminin genes (Sztal et al., 2011), in *apc* mutant, BIO-treated and heat-shocked *HS:dkk1-GFP* embryos. This quantitative RT-PCR analysis revealed that except for laminin β2l (*lamb2l*) all other laminin genes were upregulated, with *lama5* showing the maximum (1.8-fold) increase, when canonical Wnt signalling was constitutively active (Fig. 7A). By contrast, transcript levels of laminin genes, especially that of *lamα5* (0.34±0.11 fold), were significantly decreased in *HS:dkk1-GFP* embryos in response to a reduction in Wnt signalling (Fig. 7A). *In situ* hybridisation revealed highest expression of *lamα5* at the distal end and the least expression at the proximal end in the wild-type median fin fold. Interestingly, *apc* mutant and BIO-treated embryos exhibited an increase and an expansion of *lama5* expression in the fin fold (Fig. 7D-G′). By contrast, in heat shocked *HS:dkk1-GFP* embryos, the *lamα5* expression was lower as compared to the control (Fig. 7H-I′). Immunostaining followed by quantification of laminin staining intensities further corroborated these findings (Fig. 7B-C⁗). These data elucidate that the differential levels of canonical Wnt signalling sets up a graded expression of laminin α5 in the median fin fold. Our analysis further revealed that the regulation of laminin levels by canonical Wnt signalling is also seen in other locations such as the midbrain hindbrain boundary, lens, retina, otic placodes and notochord (supplementary material Fig. S7A-G′). Consistently, we observed five TCF-binding sites in the upstream regulatory region of the *lamα5* gene (supplementary material Fig. S7H; see methods in the supplementary material).

To test the hypothesis further, we asked whether epithelial cell shape regulation by Wnt signalling is dependent on laminin α5. Indeed, upon knockdown of *lamα5* in both *apc* mutant and BIO-treated embryos, the cells show relatively lower aspect ratios and were far less stretched when compared with the control embryos (Fig. 7J-M). In addition, we performed single and double knockdown of *lama5* and *itga3* in combination with BIO treatment and analysed the cell shapes at 30 hpf. Loss of either *lama5* or *itga3* (or both) in BIO-treated larvae resulted in cells with polygonal or intermediate morphology (supplementary material Fig. S6H-L). These data suggest that Wnt signalling-mediated cell stretching in the median fin epithelium is dependent on laminin α5 and integrin α3.

To understand whether the Wnt signalling specifically regulates laminins or modulates the expression of ECM components in general, we analysed the expression of *fras1*, *frem1a* and *frem2a* during fin development under gain and loss of Wnt signalling function scenario. We observed retention of expression of these three genes at the distal end and mild expansion of their expression domain in caudal-most part of the fin fold in the *apc* mutants. There was no significant difference in the expression of these genes in BIO-treated and heat-shocked *HS:dkk1-GFP* embryos (supplementary material Fig. S8A-I′). As loss of Wnt signalling does not yield any phenotype, the mild effect on expression of these ECM components in the *apc* mutant could be a secondary consequence of the altered fin morphology.

To conclude, our studies demonstrate a novel mechanism wherein the levels of canonical Wnt signalling dictate the levels of laminins, which along with integrin α3 influence cellular patterning in the developing median fin epithelium. Wnt signalling also regulates laminin levels in the brain, eyes, otic placodes, somites and notochord, suggesting a wide regulation of these ECM components by Wnt signalling. Other ECM components do not seem to be controlled by the extent of Wnt signalling during fin development.

### Canonical Wnt signalling regulates epithelial patterning of the pectoral fins

**Fig. 8.**
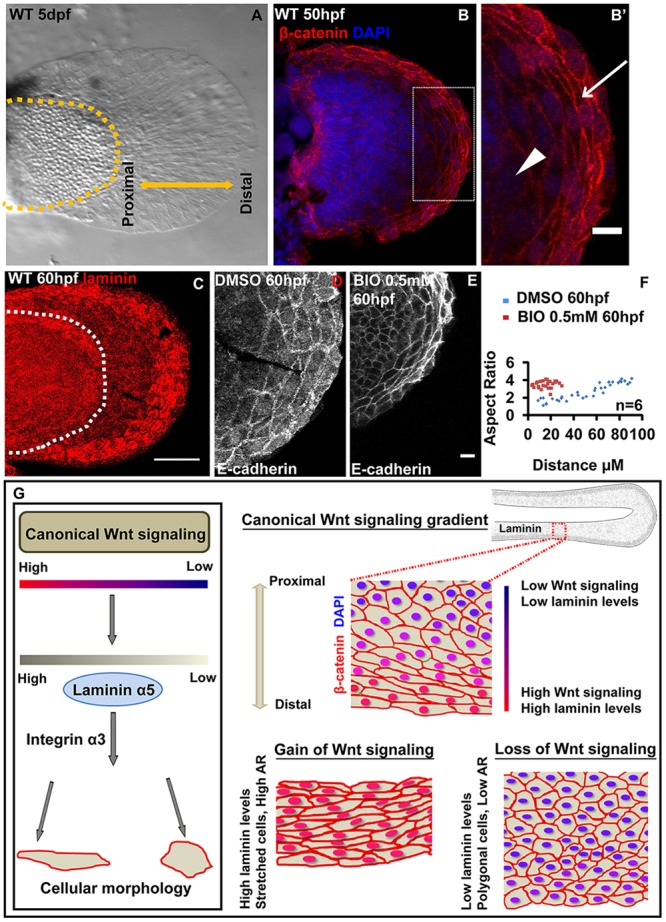
**Mechanisms patterning median fin fold epithelium are also conserved in the pectoral fin.** Bright-field image (A) of 5 dpf wild-type pectoral fin. β-Catenin and DAPI-stained pectoral fin (B) and its digitally zoomed (B′) region (box in B). Arrow in B′ indicates stretched epithelial cells at the distal end, whereas arrowhead indicates polygonal cells. (C) Maximum intensity projections of wild-type larval pectoral fin stained for laminin. Confocal images showing E-cadherin staining in DMSO- (D) or BIO-treated (E) larval pectoral fins. (F) Comparison of aspect ratios of pectoral fin epithelial cells in DMSO and BIO-treated larvae. (G) Model showing the regulation of epithelial patterning in zebrafish fin-fold epithelium by the canonical Wnt signalling gradient. A gradient of canonical Wnt activity exists along the PD axis of the fin fold. The gain- and loss-of-function analyses reveal that the extent of canonical Wnt signalling regulates the expression of the ECM component laminin α5, which directly regulates cell shapes by interacting with integrin α3. Scale bars:10 µm in B′,D,E; 50 µm in B,C. AR, aspect ratio.

Canonical Wnt signalling patterns the epithelium of zebrafish median fin (an unpaired appendage) along the PD axis. We asked whether this mechanism is conserved in patterning the epithelium of paired appendages. We analysed the pectoral fins in zebrafish larvae (Fig. 8A). Our studies revealed that there is indeed a canonical Wnt signalling gradient along the PD axis, as shown by the nuclear accumulation of β-catenin (Fig. 8B,B′). This is consistent with the expression of the Wnt reporter in the distal domain of the pectoral fin (supplementary material Fig. S9A,B; Moro et al., 2012). Correlating with this, we observed that the distal epithelial cells have a stretched morphology and high levels of laminin (Fig. 8B′,C). By contrast, cells on the proximal side have an intermediate or polygonal morphology and low levels of laminin (Fig. 8B′,C). Furthermore, the gain of Wnt signalling by BIO treatment resulted in reduction of the pectoral fin fold and all the cells along the PD axis showed a stretched morphology (Fig. 8D-F).

Our results show that the epithelia of both median and pectoral fin are patterned by canonical Wnt signalling along the PD axis, further suggesting that this developmental mechanism is conserved in both unpaired and paired fin appendages.

## DISCUSSION

Cell shape modulation is one of the major driving forces behind acquiring appropriate organ shapes during morphogenesis. Appendage morphogenesis is regulated by the concerted activities of various signalling pathways, including Wnt. Here, we have identified a novel mechanism in which canonical Wnt signalling controls laminin synthesis to regulate epithelial cell shapes and tissue morphology during vertebrate appendage development (Fig. 8G).

Wg/Wnt signalling has been shown to regulate appendage development in invertebrates as well as vertebrates (Barrow et al., 2003; Church and Francis-West, 2002; Fernandez-Teran and Ros, 2008; Galceran et al., 1999; Geetha-Loganathan et al., 2008; Kawakami et al., 2001; Kengaku et al., 1998; Martinez Arias, 2003; Neumann and Cohen, 1997; Soshnikova et al., 2003). However, it has remained unclear whether or not a gradient of canonical Wnt signalling exists in vertebrate appendages. Here, we have shown that a gradient of canonical Wnt signalling activity is present along the PD axis in median as well as pectoral fins of zebrafish (Fig. 8B′,G). Our *in situ* hybridisation analysis suggests that a graded expression of *wnt3a* and *fzd2* along the proximo-distal (PD) axis may help to establish this gradient. Unfortunately, neither *wnt3a* nor *frz2* knockdown results in the loss of epithelial pattern in the median fin fold, presumably due to the functional redundancy between Wnt ligands and receptors (M.N. and M.S., unpublished).

In *Drosophila* wing imaginal disc, the levels of Wg signalling along the PD axis directly correlates with the levels of DE-cadherin and apical constriction of the epithelial cells (Jaiswal et al., 2006). Besides, Wg signalling has also been shown to alter apical-basal length of the epithelial cells through its effects on Vestigial (Widmann and Dahmann, 2009). Our data presented here suggest that overactivation of Wnt signalling interferes in the reduction of apical-basal height of the distal fin epithelial cells and for generation of sub-epidermal space. Importantly, higher activity of canonical Wnt signalling leads to a stretched cell morphology at the distal end, whereas lower activity results in polygonal cell morphology at the proximal end of the median, as well as pectoral, fins. In contrast to *Drosophila*, we did not observe any increase in the levels of E-cadherin at the distal end, where canonical Wnt activity is maximum. However, canonical Wnt signalling has a clear effect on cell-matrix interaction in the median fin epithelial cells. Our results show that during early development, epithelial cells synthesise and secrete graded levels of laminins along the PD axis. Furthermore, our dose-dependent morpholino knockdown analysis has revealed that the levels of laminin a5 are important for cell shape acquisition in the median fin fold. The loss of integrin α3 and laminin α5 function leads to similar cell shape phenotypes, which suggests that these two genes act in the same pathway. As it is known that laminin α5 acts as a ligand for integrin α3, which mediates cell-matrix adhesion (Colognato and Yurchenco, 2000), we propose that cell stretching requires interaction of basal cells with the extracellular matrix. Our genetic analysis further suggests that Wnt signalling activity in median and pectoral fins of zebrafish controls the cell shape changes through laminin synthesis and deposition (Fig. 8G). Besides, Wnt signalling seems to regulate laminins specifically but not the other ECM components such as Fras1, Frem1a and Frem2a.

What is the significance of the cell shape patterns in the morphogenesis of the median fin fold? In fish embryos, the pectoral and median fins exhibits radial growth. In such radially growing structures, the surface area at the periphery is larger than that in the centre. Consequently, either more cells or cells stretched in a direction perpendicular to the PD axis are required to encompass this larger surface area at the periphery. Cell stretching tangential to PD axis seems to be the preferred way to encompass this increased area, as it is not only seen in the zebrafish fins (present analysis) but also reported in the growing wing imaginal disc (Legoff et al., 2013; Mao et al., 2013). It has been proposed that the tension generated by cell stretching at the periphery is important to constrain the radial growth happening at the centre (Legoff et al., 2013). Such a balanced system is important for the acquisition and maintenance of the tissue morphology. To test whether this notion is true, further investigation involving (1) more penetrant loss-of-function analysis of Wnt signalling and (2) physical, chemical or genetic manipulation of the tension generated by stretched cell at the periphery would be essential. Nevertheless, our gain of Wnt signalling analysis clearly shows that cells stretching throughout the PD axis severely alters fin morphology and compromises the formation of sub-epidermal space. Computer models (Mao et al., 2013) predict that differential cell proliferation, as well as higher friction with the substratum (cell-matrix adhesion), can generate stretched cell morphologies at the periphery of the growing wing disc. Although differential proliferation leads to generation of cell stretching perpendicular to the PD axis, higher friction results in cell stretching along the PD axis. Contrary to this friction model, our experimental evidence suggests that differential cell-matrix adhesion, mediated by laminin α5 and integrin α3 results in stretching of cells perpendicular to the PD axis in zebrafish fin epithelium. Whether differential proliferation contributes to cell stretching in the fin fold remains to be tested. However, dampening cell proliferation throughout the fin epithelium does not seem to have any effect on the cell shapes.

The *Drosophila* wing epithelium during its transition from disc to wing also exhibits cell shape changes from columnar to cuboidal, which are dependent upon integrins and ECM components such as laminin (Dominguez-Gimenez et al., 2007). Thus, in invertebrates as well as in vertebrate appendages, laminin plays a crucial role in appendage morphogenesis. To date, it has remained unclear how the synthesis of laminins is regulated during appendage development. Our data show that laminin levels are regulated by canonical Wnt signalling in the unpaired median fin, an evolutionarily ancient appendage (Fig. 8G). In fact, this regulation does not seem to be restricted to the fin epithelium but also exists in the brain, retina, lens, notochord and somites, where active tissue remodelling and morphogenesis takes place. Our work warrants further analysis of canonical Wnt signalling and laminin synthesis in other animal models to test whether such regulation is conserved during evolution. Our preliminary analysis indicates that in *Xenopus* larval median fin, canonical Wnt signalling activity is present in the distal epithelial cells, which exhibit stretched morphologies and higher laminin deposition. Furthermore, a reporter line clearly shows Wnt signalling activity at the distal end of the *Xenopus* median fin fold (Tran et al., 2010). Besides, an interesting link between canonical Wnt signalling and modulation of basement membrane components, such as laminins and fibronectin, has been shown during the formation of the mouth in *Xenopus* (Dickinson and Sive, 2009).

To summarise, our study establishes the zebrafish larval median fin fold as a simple yet powerful paradigm in which to study epithelial patterning in vertebrate appendages and to uncover ancient mechanisms used in appendage patterning. We have identified one such mechanism in which canonical Wnt signalling controls laminin synthesis and regulates cell shape changes in the fin epithelium along PD axis (Fig. 8G).

## MATERIALS AND METHODS

### Fish strains and genotyping

Fish lines used were *apc^CΑ50a^* (Paridaen et al., 2009), HS:dkk1-GFP (Stoick-Cooper et al., 2007) and *Tg(7*×*TCF-Xla.Siam:GFP)^ia^^4^* (Moro et al., 2012). For *in situ* hybridization, embryos from *albino* line or embryos from *apc^CΑ50a/+^, HS:dkk1-GFP* and Tü lines treated with 200 µM phenylthiourea were used. The phenotype of *apc^CΑ50a^* mutant at 36 hpf was identified based on heart oedema, curvature of posterior body axis and swelling of otic placodes. Genotyping of *apc^CΑ50a^* was carried out as described previously (Paridaen et al., 2009). For zebrafish maintenance and experimentation, the guidelines recommended by CPCSEA, Government of India, were followed.

### RNA isolation and quantitative/semi-quantitative RCR

Embryos were anaesthetised in MESAB (E10521 Sigma), the flank and the median fin fold was chopped and total RNA was extracted using Trizol (Invitrogen). cDNA was synthesised using Cloned AMV-RT-kit (Invitrogen). Quantitative RT-PCR was carried out in AB Step Plus RT PCR system using appropriate primers (see methods in the supplementary material). The splicing efficiency for different doses of *laminin α5* MO was estimated as described previously (Webb et al., 2007) and actin transcript levels were used as control for normalisation.

### Whole-mount immunostaining and *in situ* hybridisation

Immunostaining and *in situ* hybridisation were carried out on PFA-fixed embryos as described previously (Sonawane et al., 2009). For the details, refer to methods in the supplementary material. Sections of *in situ* hybridisation staining were carried out as described and counterstained with 1% Eosin (Sonal et al., 2014).

### Morpholino injections and inhibitors, and heat-shock treatment

The published morpholinos (GeneTools) against laminin α5 (splice morpholino; 200 µM) and integrin α3 (ATG morpholino; 400 µM) were used for microinjections (see methods in the supplementary material for morpholino sequences) in one- to two-cell stage embryos (Carney et al., 2010; Webb et al., 2007).

IWR (13659, Cayman Chemicals) and BIO (B1686, Sigma) were dissolved in DMSO (stock 50 mM). The embryos were treated with 30 µM of IWR or 5, 50 and 500 µM of BIO in E3 buffer containing 2% DMSO. For local treatment of BIO, Sephacryl S-400 (Promega) beads were used on embryos embedded in agarose (see methods in the supplementary material). Heat-shock conditions for 20 hpf *HS:dkk-GFP* embryos were a shift from 29°C to 39°C for 2 h, followed by incubation at 29°C for 1 h and another shift to 39°C for 1 h. Embryos were then fixed.

### Microscopy and image analysis

Bright-field and fluorescence imaging was carried out using Zeiss Discovery and Confocal (LSM 10) microscopes, respectively. The quantification of aspect ratios and nuclear β-catenin intensity was made using ImageJ. For the details, refer to the methods in the supplementary material.

## Supplementary Material

Supplementary Material
