# Supplementary material for: Canonical Wnt signalling regulates epithelial patterning by modulating levels of laminins in zebrafish appendages
Source: Development. 2015 Jan 15;142(2):320–30. doi: 10.1242/dev.118703 (PMC4302845; doi:10.1242/dev.118703)
Supplement: Supplementary Material [file supp_142_2_320__index.html]

Supplementary Material 

# Canonical Wnt signalling regulates epithelial patterning by modulating levels of laminins in zebrafish appendages

## DEV118703 Supplementary Material

**Files in this Data Supplement:**

- Supplementary Material
