# Supplementary material for: Canonical Wnt signalling regulates epithelial patterning by modulating levels of laminins in zebrafish appendages
Source: Development. 2015 Jan 15;142(2):320–30. doi: 10.1242/dev.118703 (PMC4302845; doi:10.1242/dev.118703)
Supplement: Supplementary Material [file supp_dev.118703_DEV118703supp.pdf]

**Supplementary methods**

**Primers used for quantitative RT-PCR of laminin transcripts**

***lamα3* FP** 5'-AGCCGTATTGAGGGCCCTTA-3'

***lamα3* RP** 5'-CCACTCGGCTTCTTCTCTCTGT-3'

***lamα5* FP** 5'-TCCACCCACAGCCACATG-3'

***lamα5* RP** 5'-TCAGGCCACTGGAGGAGTTC-3'

***lamβ1a* FP** 5'-CCAGGAAGGAGGCGTCAGA-3'

***lamβ1a* RP** 5'-TTGAGTGCCTCGGCTACAGA-3'

***lamβ2* FP** 5'-AGACGCTTCTCGACGCAACT-3'

***lamβ2* RP** 5'-GGCACGCTGACACACACAGT -3'

***lamβ2l* FP** 5'-AAGCTGGCACAGGCTCAAGA-3'

***lamβ2l* RP** 5'-AAGCGATCTCCGCTCTCAAGT-3'

***lamγ1* FP** 5'-AGCGACTACGACCGTCAGATC-3'

***lamγ1 RP*** 5'-GAGACGGCGTGTTGAAGCA-3'

***actin* FP** 5'-ATCACACCTTCTACAACGAGC-3'

***actin* RP** 5'-CATCACCAGAGTCCATCACG-3'

**Sequences of *laminin α5* and *integrin α3* morpholinos used for microinjections**

*laminin α5* MO 5'AACGCTTAGTTGGCACCTTGTTGGC-3'

*laminin α5* Control MO 5'-AACCCTTACTTCGCACGTTCTTGGC-3'

*integrin α3* MO 5'-GTGCAGAGACTTTCCGGCCATATTT-3'

*integrin α3* Control MO 5'-GTCCACAGAGTTTCCGCCGATATTT-3'

**Whole mount Immunostaining and *in situ* hybridisation**

For the detection of nuclear β-catenin with mouse anti β-catenin (C7207 Sigma), post-fixation in methanol was omitted. In the case of laminin antibody, heat based antigen retrieval protocol was followed post-fixation in methanol. The samples were downgraded to PBS, equilibrated in 150mM Tris-Cl for 10 minutes, incubated at 70°C for 15 minutes followed by 50% acetone in PBS, absolute acetone and kept at -20°C for 20 minutes. Then the samples were downgraded to PBS and processed as mentioned above. Primary antibodies were diluted as follows: anti β-catenin (C7207 Sigma;1:500); anti phospho-Histone3 (06-570 Millipore;1:200); E-cadherin (610182 BD-Transduction Labs;1:100); Laminin (L9393 Sigma;1:100). Cy3, Alexa 488 conjugated anti-mouse or anti-rabbit secondary antibodies (Jackson Immunoresearch Labs; Molecular Probes) were used. In some cases DAPI (stock concentration 10mg/ml; working dilution 1:100) was used as a counter-stain.

For making *In situ* hybridisation probes, the following were cloned: *lamα5* (NM_001039171.1; 10337-11077 nucleotides), *wnt3a* (NM_001007185.1; 52-786 nucleotides), *fras1* (NM_001130840.1; 9683-10349 nucleotides), *frem1a* (NM_001190308.1; 903-1516 nucleotides) and *frem2a* (NM_001137657.1; 8669-9170 nucleotides) and probes were synthesised using DIG labelling kit (Roche).

**Local treatment of BIO using Sephacryl beads**

Sephacryl S-400 (Promega) beads were washed in ethanol and soaked in 50mM BIO or only DMSO with gentle shaking. At 21hpf, zebrafish larvae were immobilised in 0.8% low gelling agarose, a portion of the agarose above the ventral fin was removed and about 5-10 beads soaked in BIO or in DMSO were placed and gently pushed down. The immobilised larvae were covered with E3 buffer, incubated at 29°C and analysed at 29hpf.

**Identification of potential TCF binding sites**

For the identification of TCF binding sites in *laminin α5,* 5500bp sequence upstream to the start codon was analysed for the presence of consensus sites - CTTTG-A/T-A/T (Tetsu et al.,1999); T/A-T/A-CAAAG (Bottomly et al.,2010); CTTTG-A/T-C/G (Yochum et al.,2011). *axin2,* a known downstream target of canonical Wnt signalling was used as a positive control.

**Microscopy and image analysis**

For bright field imaging, the embryos were anaesthetised with MESAB and mounted in 3% methyl cellulose. Immunostainings were imaged in a 12bit format using a Zeiss Confocal microscope (LSM 510) at 40X oil and 10X magnifications. The pinhole values were kept as 1 Airy Unit to give optical sections of about 0.44µm thickness. Images were taken in the ventral part of the median fin fold with the 40X oil objective at 1x zoom having 224.78µm x 224.78µm dimensions in the x-y plane. *In situ* hybridisation staining were imaged on Zeiss Discovery using a 1X objective lens.

To measure aspect ratios, cell outline was drawn in ImageJ, the software fits an ellipse and gives the values of major and minor axes; their ratios were calculated using MS-

Excel. To measure β-catenin levels, a circle of fixed dimension was drawn on the chosen nuclei, the intensities of β-catenin (in Red) and DAPI (in Blue, for normalisation) were measured for 3 prominent image slices per cell and the means were estimated. The distance of the corresponding cell is measured from the base of the fin fold along the PD axis. The data was plotted as aspect ratios or nuclear β-catenin intensities along the Y-axis and the corresponding nuclear distances along the X-axis. Laminin intensities were measured on maximum intensity projections, a circle of fixed dimension was drawn at various points in a line along the PD axis, the corresponding intensity and distance was measured and plotted. For analysing cell shapes in cross sectional view we used ImageJ to obtain orthogonal sections of confocal images.

**Supplementary Figures**

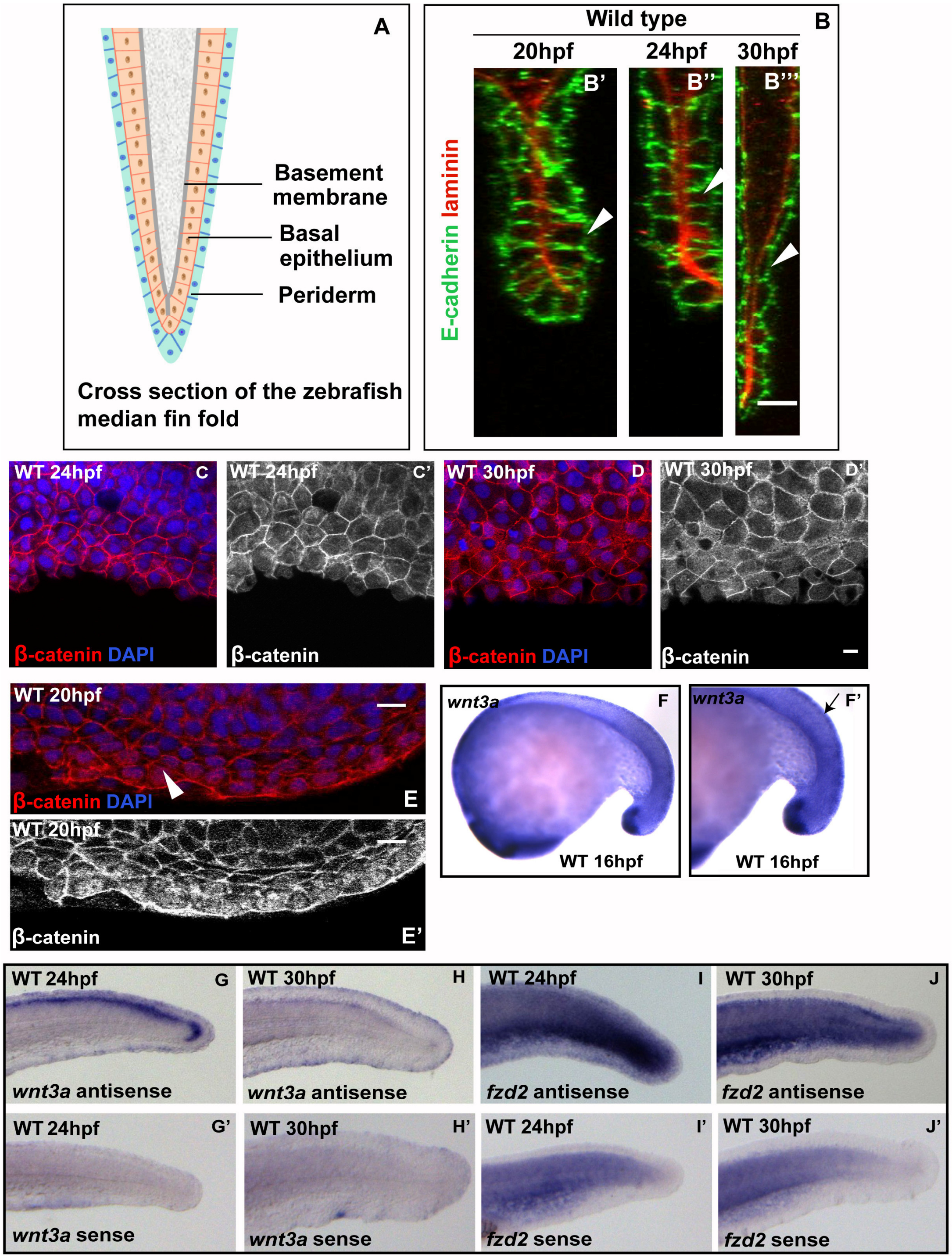


**Figure S1.** Illustration of the cross sectional view of median fin epithelium (A) showing epithelial fold consisting of basal epithelium (epidermis) and periderm.

Orthogonal sections along the PD axis of fin (B) showing cells shapes in cross-sectional view at 20 (B'), 24 (B'') and 30hpf (B'''). Confocal images of the median fin peridermal cells at 24 and 30hpf (C-D') and basal epidermal cells at 20 hpf (E,E') in wild type embryos showing overlay of β-catenin and DAPI (C,D, E) and only β-catenin staining (C',D',E'). *In situ* hybridisation using *wnt3a* probes in wild type embryos at 16 hpf (F). A magnified view with arrows to indicate *wnt3a* expression in the developing median fin (F'). *In situ* hybridisation in wild type embryos at 24 and 30hpf for *wnt3a* (G, H) and *fzd2* (I,J) as well as their respective sense controls (G'-J'). Arrowhead in E indicates nuclear β-catenin. Scale bar represents 10 µm in (B-E').

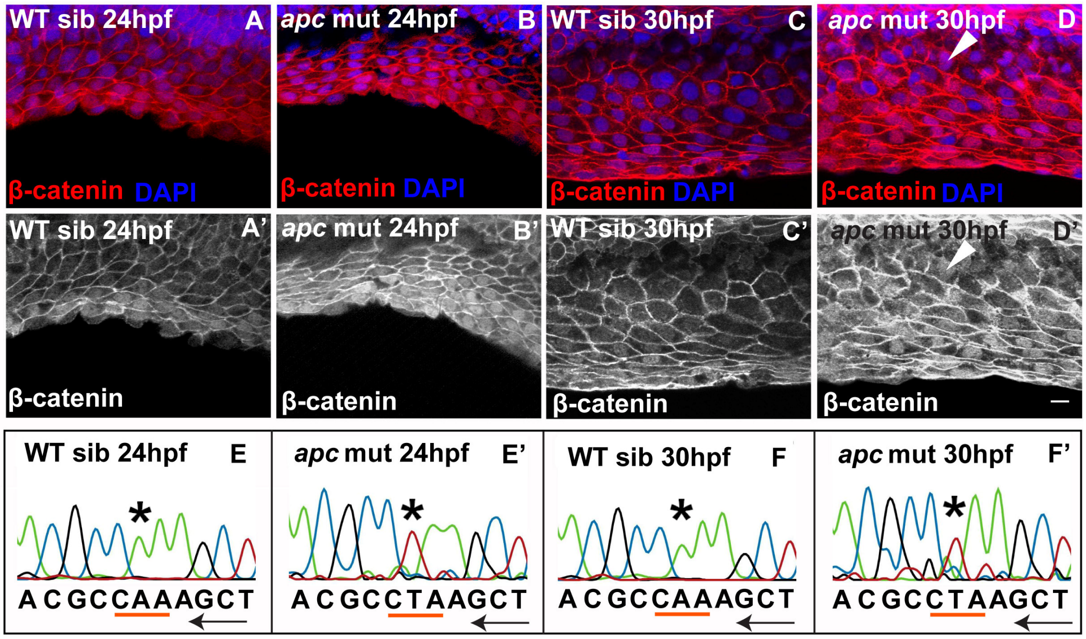


**Figure S2.** Confocal images of the fin fold in 24 and 30 hpf wild type sibling and *apc* mutant embryos showing overlay of β-catenin and DAPI (A-D) and only β-catenin staining (A'-D'). Chromatograms of the genotyped WT sibling and *apc* mutant at 24 and 30hpf (E-F'). Black arrows indicate that sequence is from reverse strand. Arrowheads in D and D' indicate nuclear β-catenin in proximal cells. Scale bar represents 10 μm in A-D'.

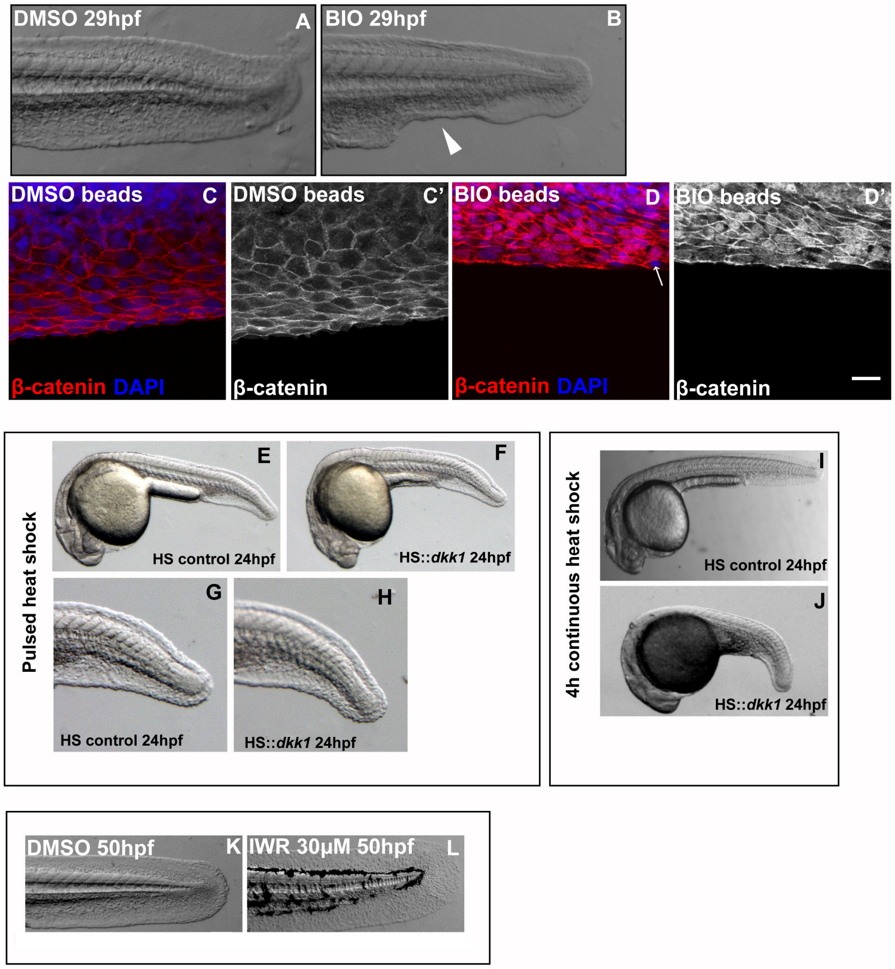


**Figure S3.** Bright field images of the median fin fold incubated with sephacryl beads loaded with DMSO (A) and with BIO (B). Confocal images of the fin fold at 29hpf wild type embryo incubated with DMSO loaded (C,C') and BIO drug (D, D') loaded beads showing overlay of β-catenin and DAPI (C, D) and only β-catenin staining (C', D'). Bright field images of HS Control and *HS:dkk1-GFP* embryos either with pulsed heat shock for 3hours (at lower magnification; E,F and at higher magnification; G,H) or continuous heat shock for 4 hours (I,J) between 20hpf to 24hpf. DMSO (K) and IWR (L) treated embryos at 50hpf. Arrowhead in B shows reduction in fin area corresponding to the region where beads soaked in BIO were placed. Arrow in D indicates the nuclei of a peridermal cell. Scale bar represents 10 μm in C-D'.

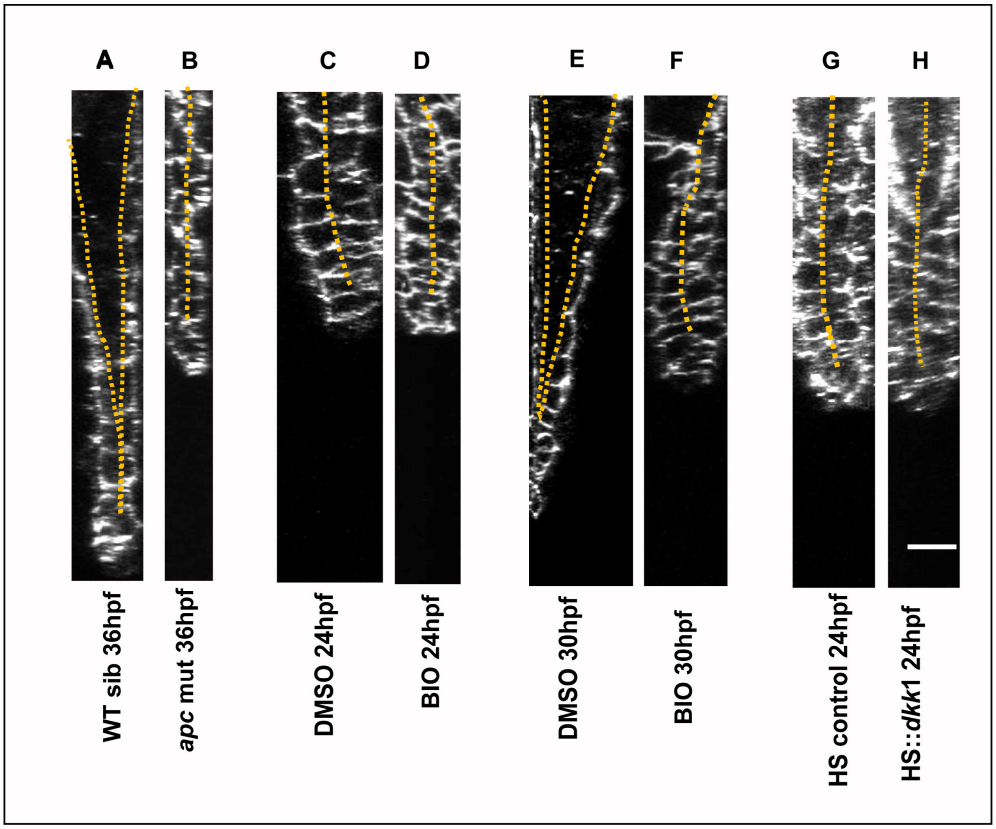


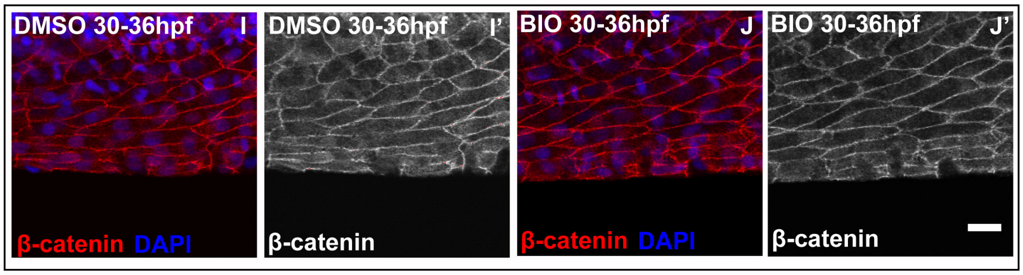


**Figure S4.** Orthogonal sections of confocal images showing cross sectional view of cell shapes across the PD axis of median fin stained for E-cadherin in wild-type sibling (A) and *apc* mutant (B) at 36hpf; DMSO (C,E) and BIO treated (D,F) at 24 and 30hpf; HS Control (G) and *HS:dkk1-GFP* (H) at 24hpf. Confocal images of the median fin in wild type embryos treated with DMSO and BIO at 30hpf and analysed at 36hpf showing overlay of β-catenin and DAPI (I, J) and only β-catenin staining (I', J'). Scale bar in H and J' equals 10 μm in A-H; I-J'.

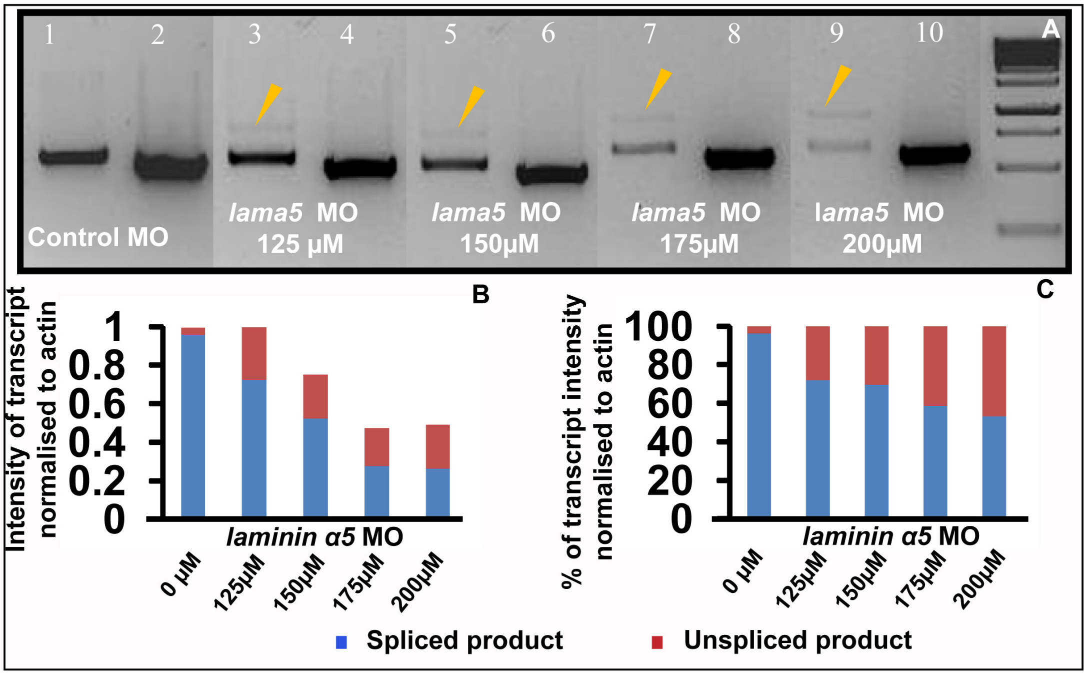


**Figure S5**. (A) Gel images of PCR to check the levels of spliced and un-spliced transcripts (Lanes 1,3,5,7,9) upon injecting control MO and different doses *lamα5* MO. Arrowheads indicate un-spliced transcript. Actin controls are in lanes 2,4,6,8,10. Intensity plots (B) and percentage intensity plots (C) of spliced and un-spliced transcript levels normalised to actin in control MO and *lamα5* MO injected embryos.

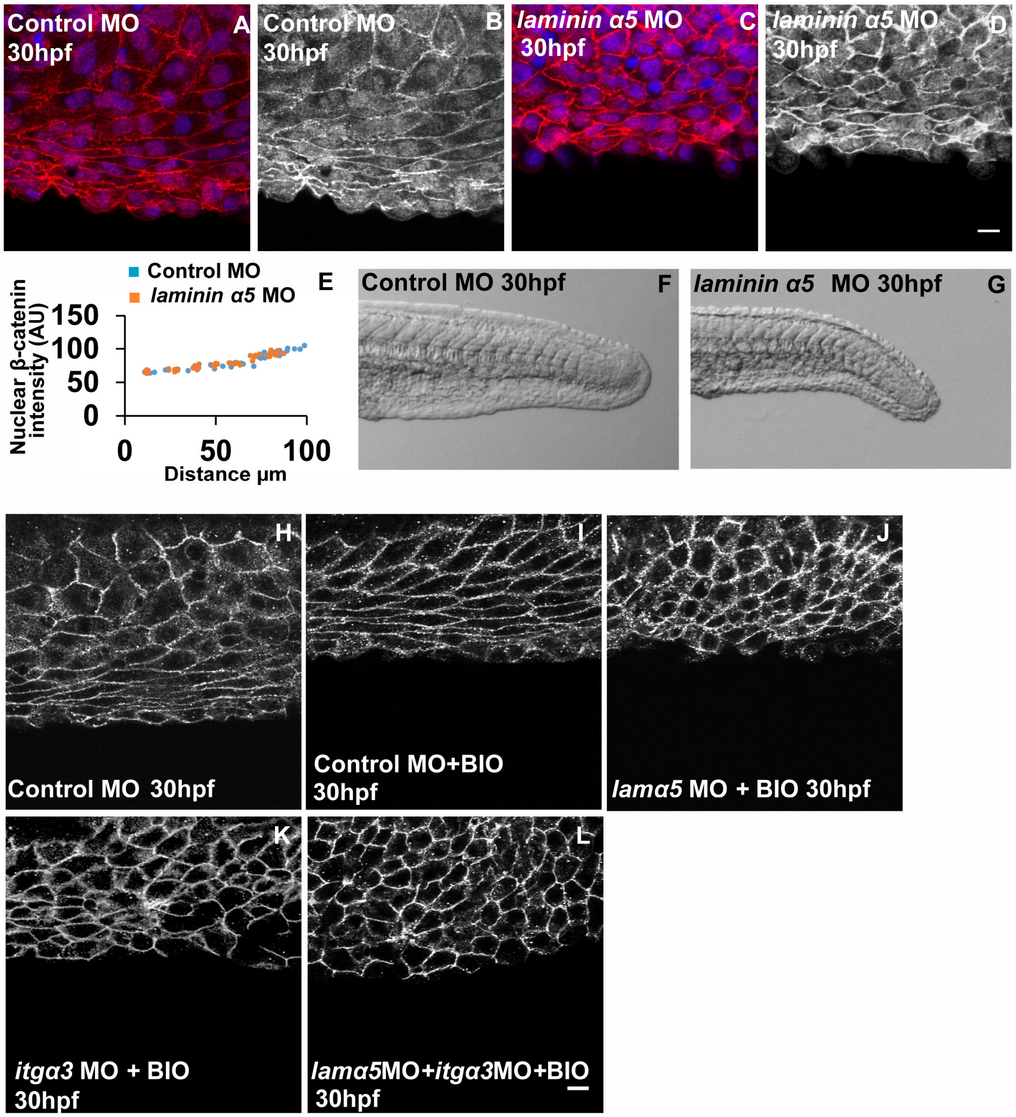


**Figure S6**. Confocal images of median fin in control MO (A,B) and *lamα5* MO (C,D) injected embryos at 30hpf showing an overlay of β-catenin and DAPI (A,C) and only β-catenin staining (B,D) and intensity plot comparing their nuclear β-catenin levels (E). Bright field images of 30hpf control (F) and *lamα5* (G) morphants. E-cadherin stainings of control MO injected (H), control MO injected and BIO treated (I), *lamα5* MO injected and BIO treated (J), *itgα3* MO injected and BIO treated (K) *lamα5, itgα3* double MO injected and BIO treated (L) fin fold epithelial cells. Scale bars in D and L corresponds to 10 µm in A-D; H-L. (AU- Arbitrary Units).

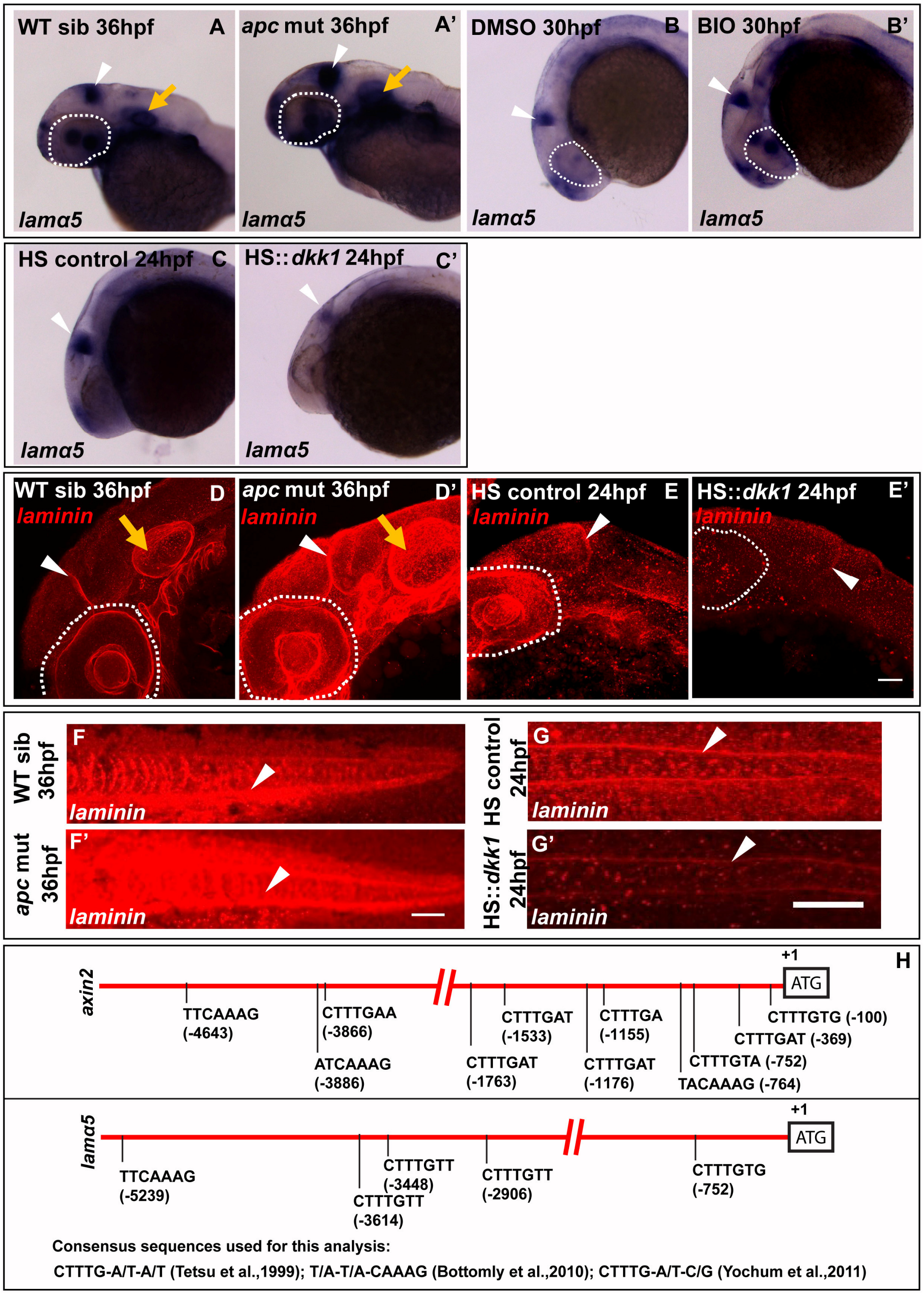


**Figure S7**. *In situ* hybridisation using *lamα5* probes in sibling (A), *apc* mutant (A'), DMSO control (B), BIO treated (B'), heat shock control (C) and heat shocked *HS::dkk-GFP* embryos (C'). Confocal images showing laminin imunostaining in

sibling (D,F), *apc* mutant (D',F'), heat shock control (E,G) and heat shocked *HS::dkk-GFP* embryos (E',G'). A schematic showing Tcf binding sites in *axin2* and *lamα5* promoters (H). Arrowheads in A-C' and D-E' points to midbrain-hindbrain boundary (MHB) while arrows in A, A' and D, D' indicate otic placodes. Arrowheads in F-G' indicate laminin staining in notochord. Dotted lines demarcate the eye. Scale bars in E', F' and G' correspond to 50 μm in D-G'.

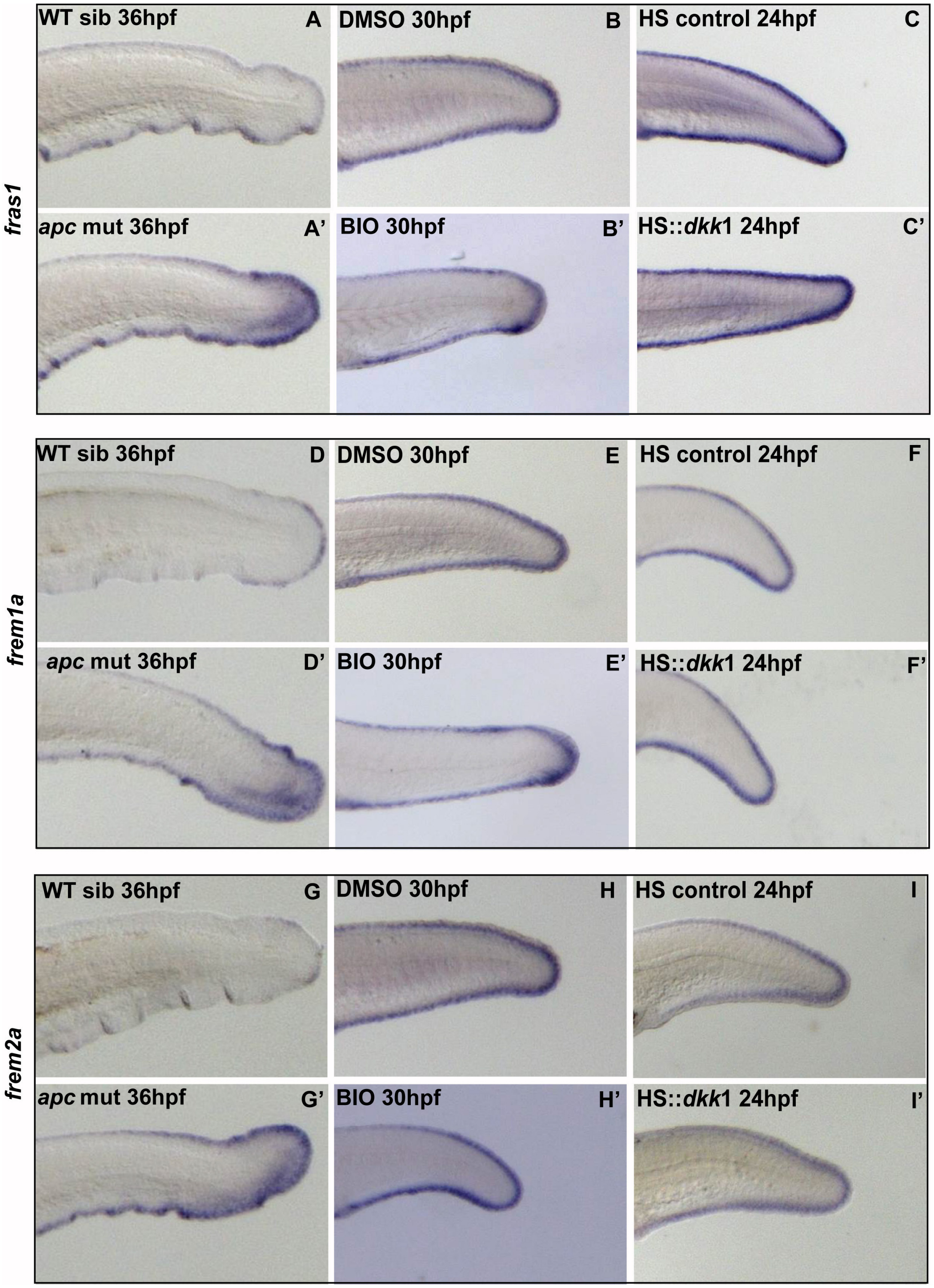


**Figure S8**. *In situ* hybridisation using *fras1, frem1a* and *frem2a* probes in sibling (A,D,G), *apc* mutant (A',D',G'), DMSO control (B,E,H), BIO treated (B',E',H'), heat shock control (C,F,I) and heat shocked *HS::dkk-GFP* embryos (C',F',I').

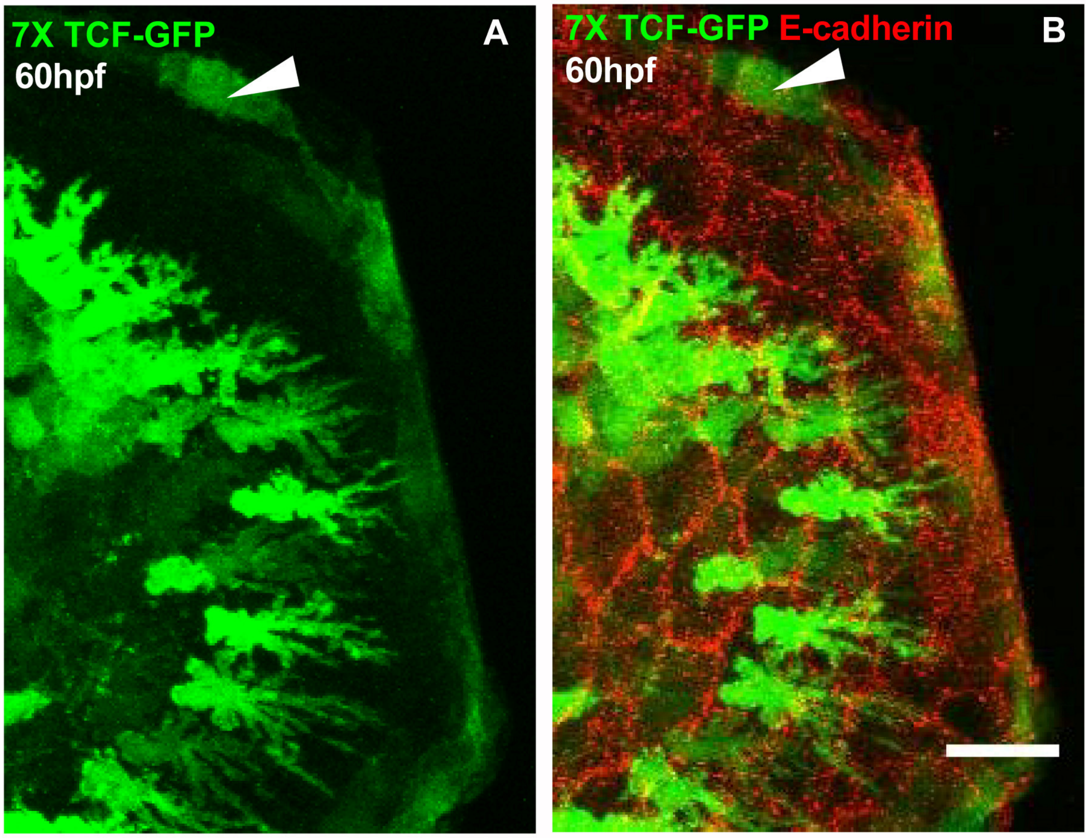


**Figure S9**. Confocal images of pectoral fin in *Tg(7xTCF-Xla.Siam:GFP)*$^{ia4}$, a Wnt reporter line showing only GFP expression (A) and overlay of GFP with E-cadherin staining. Arrowheads indicate a distal stretched cell with high levels of GFP. Scale bar in B corresponds to 10 µm.
